# Age, Gender and Season Are Good Predictors of Vitamin D Status Independent of Body Mass Index in Office Workers in a Subtropical Region

**DOI:** 10.3390/nu12092719

**Published:** 2020-09-05

**Authors:** Li-Kai Wang, Kuo-Chuan Hung, Yao-Tsung Lin, Ying-Jen Chang, Zhi-Fu Wu, Chung-Han Ho, Jen-Yin Chen

**Affiliations:** 1 Department of Anesthesiology, Chi Mei Medical Center, Tainan 71004, Taiwan; anesth@gmail.com (L.-K.W.); ed102605@gmail.com (K.-C.H.); anekevin@hotmail.com (Y.-T.L.); 0201day@yahoo.com.tw (Y.-J.C.); aneswu@gmail.com (Z.-F.W.); 2 Department of Health and Nutrition, Chia Nan University of Pharmacy and Science, Tainan 71710, Taiwan; 3 Center of General Education, Chia Nan University of Pharmacy and Science, Tainan 71710, Taiwan; 4 College of Health Sciences, Chang Jung Christian University, Tainan 71101, Taiwan; 5 Department of Anesthesiology, Tri-Service General Hospital and National Defense Medical Center, Taipei 11490, Taiwan; 6 Department of Medical Research, Chi Mei Medical Center, Tainan 71004, Taiwan; ho.c.hank@gmail.com; 7 Department of the Senior Citizen Service Management, Chia Nan University of Pharmacy and Science, Tainan 71710, Taiwan

**Keywords:** occupational health, hypovitaminosis D, hypocalcemia, subtropical, season, gender, age

## Abstract

This study aimed at determining the prevalence and predictors of hypovitaminosis D (serum 25-hydroxyvitamin D < 30 ng/mL) among office workers in a subtropical region from an electronic hospital database. Totally, 2880 office workers aged 26–65 years who received health examinations with vitamin D status and total calcium concentrations at a tertiary referral center were retrospectively reviewed. Subjects were divided into groups according to genders, age (i.e., 26–35, 36–45, 46–55, 56–65), body-mass index (BMI) (i.e., obese BMI ≥ 30, overweight 25 ≤ BMI < 30, normal 20 ≤ BMI < 25, and underweight BMI < 20) and seasons (spring/winter vs. summer/autumn) for identifying the predictors of hypovitaminosis D. Corrected total calcium level <8.4 mg/dL is considered as hypocalcemia. Multivariate logistic regression demonstrated that females (AOR 2.33, (95% CI: 1.75, 3.09)), younger age (4.32 (2.98, 6.24), 2.82 (1.93, 4.12), 1.50 (1.03, 2.17)), and season (winter/spring) (1.55 (1.08, 2.22)) were predictors of hypovitaminosis D, whereas BMI was not in this study. Despite higher incidence of hypocalcemia in office workers with hypovitaminosis D (*p* < 0.001), there was no association between vitamin D status and corrected total calcium levels. A high prevalence (61.9%) of hypovitaminosis D among office workers in a subtropical region was found, highlighting the importance of this occupational health issue.

## 1. Introduction

Vitamin D is a secosteroid hormone regulating calcium homeostasis and bone metabolism. During the past decades, associations between vitamin D status and extra-skeletal health have been recognized. Vitamin D status is highly associated with the risks of autoimmune diseases [1], metabolic syndrome [2,3] and neuropathic pain [4,5]. Serum 25-hydroxyvitamin D (25(OH)D) is generally used as a marker of vitamin D status [6,7]. Based on the 2011 Endocrine Society Clinical Practice Guidelines, vitamin D status is defined as optimal (i.e., serum 25(OH)D concentration: 30–150 ng/mL) and suboptimal (i.e., serum 25(OH)D concentration <30 ng/mL, hypovitaminosis D). Hypovitaminosis D includes insufficiency and deficiency defined as serum 25(OH)D concentrations 20–30 ng/mL and <20 ng/mL, respectively [8].

Vitamin D is called the “sunshine vitamin.” During exposure to sunlight, ultraviolet-B (UVB) stimulates the conversion of 7-dehydrocholesterol (provitamin D) in the skin to pre-vitamin D which in turn isomerizes into vitamin D. Many factors have been shown to affect vitamin D status, including both environmental and personal characteristics/behaviors. Environmental factors, such as latitude [6] and season [9], determine whether there is sufficient UVB radiation to stimulate dermal vitamin D synthesis. Personal factors including gender [10,11,12], age [6,13], race [14,15], adiposity [15,16,17], physical activity [18], dietary habits [12,13] and occupation [19] can influence individual vitamin D status. Serum vitamin D status is a complex result of environmental and personal factors [6,20].

Indoor workers are consistently reported as being the occupational group most likely to suffer from hypovitaminosis D due to limited exposure to UVB radiation [19]. Subtropical regions are located between the tropic and temperate zones. Subtropical regions have plenty of sunshine all year round. Thus, people living in subtropical regions should not have hypovitaminosis D status. A hypovitaminosis D status was found in 44.1% of the adults aged 65 and older in a subtropical region and season was not a predictor of hypovitaminosis D in the study population [21]. Despite the common assumption that people living in sunny countries are at low risk of hypovitaminosis D, hypovitaminosis D was found to be highly prevalent among office workers in subtropical Australia [9]. Season was found to be more important than age and gender in determining serum 25(OH)D level for office workers in subtropical Australia [9]. Intriguingly, the association of age with serum 25(OH)D concentrations was found to vary during three study periods (i.e., 1988–1994, 2001–2004, 2007–2010) based on the data from the National Health and Nutrition Examination Survey in US [14,22]. While the serum 25(OH)D level decreased with advancing age during the first survey (i.e.,1988–1994), the level of the aged population became comparable to the younger age group during the second (i.e., 2001–2004) survey and even higher than their younger counterparts during the third survey (i.e., 2007–2010).The findings, therefore, highlighted the possible influence of other confounders rather than age per se on serum 25(OH)D levels. Furthermore, females often have a higher prevalence of hypovitaminosis D than that in males [10,11,12]. Anthropometrically, body mass index (BMI) has been found to be inversely associated with serum 25(OH)D levels [15,16,17]. Nevertheless, although Asians are known to have a lower BMI [23], the impact of BMI on serum 25(OH)D levels in Asian office workers has not been addressed.

Taking into account the scarcity of research regarding serum vitamin D status among indoor office workers in subtropical regions [9] and the fact that serum vitamin D status is the result of complex interactions among various environmental and personal factors [6,20], the present study aimed at investigating the prevalence of hypovitaminosis D among indoor office workers in subtropical Taiwan and identifying factors that independently determine 25(OH)D levels of this population. Determination of serum calcium levels is often available and widely included as a routine laboratory test, but determination of serum 25(OH)D levels may be not. It raised a question whether hypovitaminosis D could be suspected based on calcium levels. We therefore assessed the association between corrected total serum calcium levels and 25(OH)D concentrations in the study subjects.

## 2. Methods

### 2.1. Study Population

Self-administered questionnaires were used to collect data including job types (office workers, … and others) on all adults who received health examinations at Chi Mei Medical Center, a 1200-bed tertiary referral center in southern Taiwan with 22.5–22.9° N latitude. Colonoscopy, serum 25(OH)D and total calcium concentrations are grouped as one option among the health examination survey at Chi Mei Medical Center. Health examination data at Chi Mei Medical Center between 1 December 2016 and 31 November 2018 stored in the electronic database of the institute were retrospectively reviewed and analyzed. Inclusion criteria for the present study were (1) subjects aged 26–65 years (26 is the average age of Taiwanese adults with a stable occupation; 65 is the official retirement age); (2) office workers; and (3) those with available data of serum 25(OH)D and total calcium/phosphate/creatinine examinations during the study period. Exclusion criteria were: (1) individuals who had diagnostic codes of human immunodeficiency virus infection [International Classification of Diseases, Ninth Revision (ICD-9) 042, 043, 044], organ transplants (ICD-9 3751, 1160, 1164, 1169, 5059, 5280, 5283, 5569, 3350–3352), liver disease (ICD-9 571) and/or chronic renal failure (ICD-9 585)on dialysis, which are potential confounders of hypovitaminosis D [24,25,26] as well as (2) individuals whose medical records showed no evidence of serum 25(OH)D and total calcium examinations during the study period. The study was conducted in accordance with the Declaration of Helsinki.

This retrospective study was approved by the Institutional Review Board of the Chi Mei Medical Center, Tainan, Taiwan (IRB-10711-001).

### 2.2. Study Parameters and Definitions

Because the prevalence of vitamin D deficiency has been found to be statistically associated with age, gender, and seasons [27], the impacts of gender, age, season, and BMI on serum 25(OH)D concentrations in the study population were studied. To investigate the influence of age on serum 25(OH)D levels, all eligible subjects were divided into four age groups: 26–35, 36–45, 46–55, and 56–65. The seasons were categorized into the two seasons with shorter daylight (i.e., Winter and Spring) and the other two with longer daylight (i.e., Summer and Autumn) to assess the association between season and serum 25(OH)D levels. BMI was calculated as the weight in kilograms divided by the square of height in meters (kg/m^2^). All subjects were divided into those who were obese (i.e., BMI ≥ 30), overweight (25 ≤ BMI < 30), normal (20 ≤ BMI < 25), and underweight (BMI < 20) [15] to evaluate the effect of BMI on serum 25(OH)D concentrations. Corrected total serum calcium concentrations was calculated by measured total calcium+ (0.8 × (4.0−(albumin)). A serum calcium <8.4 mg/dL with a normal serum albumin (3.4 to 5.4 g/dL) is defined as hypocalcemia [28]. Hypercalcemia is considered if the corrected total serum calcium level is >10.4 mg/dL [29]. Furthermore, phosphate can bind calcium avidly to cause hypocalcemia during acute hyperphosphatemia. In chronic renal failure, hypocalcemia is common because of reduced renal synthesis of 1,25-dihydroxyvitamin D and an increase in fibroblast growth factor 23 [30]. Thus, serum phosphate levels and creatinine-based estimated glomerular filtration rate (eGFR) were also investigated.

### 2.3. Subject Selection

The study subjects were selected using block randomization approach for controlling the potential confounding factors. Because at least 30 subjects are needed for establishing a relationship in correlational research [31], a total of 60 subjects with a female to male ratio of 1:1 and at least one subject in each year age to achieve age equality for each of the four age groups were randomly selected and allocated to the 12 months of a year (i.e., from January to December) during the two-year study period so that there were 720 individuals (i.e., 360 females and 360 males) in each age group and in each season. Based on an estimated total number of 20 thousand subjects undergoing health examination during the study period, a sample size of about 10% (i.e., two thousand) would fit the recommendation for a descriptive research [31].

### 2.4. Blood Collection and Determination of Serum 25(OH)D Levels

The serum sample, which was acquired from a fasting blood sample of each subject after centrifugation and stored at a temperature of −70 °C, was subject to serum 25(OH)D quantification using automated Chemiluminescent Microparticle Immuno Assay [5,32] (ARCHITECTi2000 (Abbott, Chicago, IL, USA) as our previous study [5].

### 2.5. Blood Collection and Determination of Total Serum Calcium, Phosphate, Creatinine and Albumin

The determination of total serum calcium concentrations was based on calcium ions (Ca2+) reacting with Arsenazo III (ARCHITECT calcium reagent) to generate a purple colored complex with a sensitive absorbance peak at 600–660 nm. The absorbance of the Ca-Arsenazo III complex was measured bichromatically at 660/700 nm (ABBOTT Architect c8000/c16000). The absorbance of the Ca-Arsenazo III complex is directly proportional to the calcium concentration in the sample. In this method, magnesium does not significantly interfere with calcium determination. However, very small amounts of copper ions may interfere with calcium determination. This problem was overcome by adding 15 mmol/L thiourea to the calcium reagent [33]. Serum calcium is stable for up to 7 days at room temperature (15–25 °C) [33]. Using UV-spectrophotometry, phosphate may be determined in serum as the phosphomolybdic acid complex, the absorbance of which was measured bichromatically at 700/880 nm (ABBOTT Architect c8000/c16000). The absorbance of the phosphomolybdic acid complex is directly proportional to the phosphate concentration in the sample [34]. Serum creatinine was measured by a Jaffe method, alkaline picrate kinetic (ABBOTT Architect c8000/c16000).

Serum albumin was measured with dye-binding assay using bromocresol green (BCG) method. BCG binds albumin to form albumin-BCG complex (a colored compound). Its absorbance, measured at 630 nm (620–640), is directly proportional to the albumin concentration in the sample (ABBOTT Architect c8000/c16000) [35].

All of the aforementioned blood examinations were performed every weekday.

### 2.6. Statistical Analysis

Data processing and statistical analysis were performed using SAS statistical software (Version 9.4; SAS Institute, Cary, NC, USA). The difference of continuous data between groups was conducted by Student t test. Chi-square test or Fisher exact test was used to determine the significance of differences in categorical variables among groups. Univariate logistic regression analysis was used to identify predictors of hypovitaminosis D. Confounding factors including gender, age groups, and season were adjusted to evaluate the odds ratio in different models. Variables that were associated with hypovitaminosis D (*p* < 0.10) on univariate analyses were entered into a multivariate logistic regression model. Independent predictors of hypovitaminosis D (<30 ng/mL) were presented as adjusted odds ratios (AOR) and 95% confidence intervals (CI). A two-sided *p* value < 0.05 was deemed significant.

Corrected total serum calcium concentrations was calculated by measured total calcium + (0.8 × (4.0−(albumin))). According to corrected total serum calcium concentrations, subjects were dichotomized into two groups: hypocalcemia (<8.4 mg/dL) and normal levels (8.4~10.4 mg/dL). For identifying the sensitivity and specificity in predicting 25(OH)D, a receiver operating characteristic (ROC) curve was plotted. The area under the ROC curve (AUC) was used to measure the diagnostic ability of hypovitaminosis D in four models. Model 1: The ROC curve for hypocalcemia to predict the incidence of hypovitaminosis D. Model 2: Adjusted for age, gender and season. Model 3: Adjusted for age, gender, season and phosphorus levels. Model 4: Adjusted for age, gender, season, phosphorus levels and eGFR.

## 3. Results

### 3.1. Demographic and Anthropometric Characteristics of the Study Population

Of a total of 20,023 eligible subjects, 2880 indoor office workers (14.4%) were randomly selected from the electronic medical database. The demographic and anthropometric characteristics of the study population are shown in Table 1. Hypovitaminosis D was prevalent, with a female prominence in gender, all age groups and seasons (all *p* < 0.001). High prevalence of hyperphosphatemia (>6.5 mg/dl) was noted in the study population.

### 3.2. Distribution of Serum Vitamin D among Indoor Office Workers

**Figure 1:**
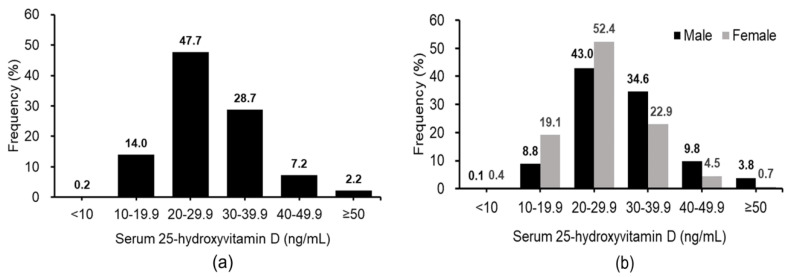
Distribution of serum 25(OH)D concentrations among indoor workers. (**a**) Age-specific distributions of serum 25(OH)D concentrations in the total study population. (**b**) Age-specific distributions of serum 25(OH)D concentrations in males and females separately.

Hypovitaminosis D (deficiency/insufficiency) was found in up to 61.9% (1782) of the study population, with deficiency (<20 ng/mL) and insufficiency (20–30 ng/mL) being noted in 14.2% (408) and 47.7% (1374) of subjects, respectively (Figure 1a). Analysis of the distribution of serum 25(OH)D concentrations demonstrated a dominance of vitamin D insufficiency in both genders (52.4% females and 43.0% males) (Figure 1b). In addition, hypovitaminosis D was significantly more common in female than in male indoor workers (71.9% vs. 51.9%, respectively, *p* < 0.001). None of the subjects showed vitamin D toxicity (i.e., serum 25(OH)D >150 ng/mL).

### 3.3. Associations of Risks of Hypovitaminosis D with Gender, Age, Season, and Body-Mass Index

The correlations between the risks of hypovitaminosis D and demographic/environmental factors (i.e., gender, age, season, and body-mass index) of 2880 indoor workers are presented in Table 2. The mean 25(OH)D concentration of the indoor workers was 28.61 ± 8.92 ng/mL. Univariate logistic regression revealed a female predominance of hypovitaminosis D (crude OR 2.37, 95% CI 2.03, 2.77). There was a significant trend of increasing incidences of hypovitaminosis D with decreasing ages with the incidence lowest in the elderly age group (56–65) and highest in the youngest age group (26–35) (*p* < 0.001) (Table 2). With reference to that of the 56–65 age group, the crude OR of hypovitaminosis D for the 26–35, 36–45, and 46–55 age groups were 3.65 (95% CI 2.91, 4.56), 2.81 (95% CI 2.26, 3.49), and 1.52 (95% CI 1.23, 1.87), respectively. There was also a significant seasonal variation in hypovitaminosis D with an increased risk in winter/spring compared to that in summer/autumn (crude OR 1.43, 95% CI 1.23, 1.67). On the other hand, there were no significant associations between BMI and hypovitaminosis D. Multivariate logistic regression analysis identified age, gender and season significant predictors of hypovitaminosis D independent of BMI in indoor office workers in subtropical Taiwan.

### 3.4. Subgroup Analysis by Genders for Predictors of Hypovitaminosis D

According to subgroup analysis by gender, multivariate logistic regression revealed that only the 36–45 and 26–35 age groups remained significant predictors of hypovitaminosis D with reference to the 56–65 age group in females (AOR 3.22; 4.05) and males (AOR 2.30; 4.73). Interestingly, season did not independently predict hypovitaminosis D in females by multivariate logistic analysis (Table 3). In males, there was a significant increase in the risk of hypovitaminosis D in winter/spring compared to that in summer/autumn by multivariate logistic analysis (AOR 1.99, 95% CI 1.40, 2.83) (Table 4). No significant associations between BMI and serum 25(OH)D were found in both genders.

### 3.5. Associations between Hypovitaminosis D and Corrected Total Serum Calcium Concentrations

**Figure 2:**
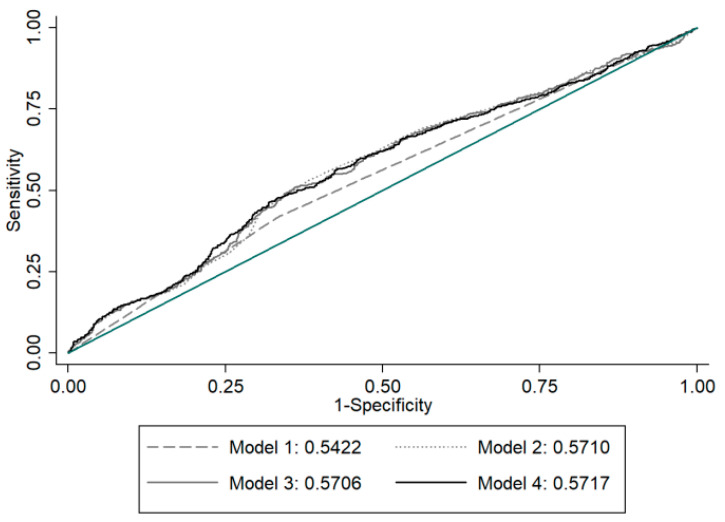
The receiver operating characteristic (ROC) curves for hypocalcemia to predict the incidence of hypovitaminosis D in Model 1. Adjusted for age, gender and season in Model 2. Adjusted for age, gender, season and phosphorus in Model 3. Adjusted for age, gender, season, phosphorus and estimated glomerular filtration rate (eGFR) in Model 4. The diagonal green line represents the line of no-discrimination.

Only one subject had a low albumin level (2.8 g/dL). Corrected total calcium concentrations of the present study population ranged from 7.06 to 10.2 mg/mL with none having hypercalcemia. Subjects with hypocalcemia had higher incidences of hypovitaminosis D (65.8%) and lower mean concentrations (8.39 mg/mL) of corrected total serum calcium levels compared to those (58.1%, 8.47 mg/mL) in subjects with an optimal vitamin D status (both *p* < 0.001) (Table 5). However, 47.5% (847/1782) subjects with hypovitaminosis D had normal corrected total serum calcium concentrations; conversely, 44.0% subjects (483/1098) with optimal vitamin D status had hypocalcemia. The ROC curves for hypocalcemia to predict the incidence of hypovitaminosis D were plotted in model 1. The AUC was 0.542 indicating no discrimination. In addition, the adjusted AUC was approximately 0.57 in Model 2, 3 and 4 indicating no discrimination (Figure 2).

## 4. Discussion

The current study demonstrated a high prevalence (61.9%) of hypovitaminosis D (deficiency/insufficiency) with 14.2% deficiency and 47.7% insufficiency among indoor office workers in subtropical Taiwan. Study on the distribution of serum 25(OH)D concentrations revealed the highest proportion of vitamin D insufficiency (i.e., 20–30 ng/dL) in both genders (52.4% females and 43.0% males) of indoor workers. The findings were consistent with those of a previous report [9] that demonstrated a high prevalence of hypovitaminosis D in indoor workers even in subtropical sunny Australia when skin exposure to UVB sunlight was limited by the nature of occupation.

Multivariate logistic regression showed that age, gender and season were significant predictors of hypovitaminosis D in indoor workers independent of BMI in subtropical Taiwan. Interestingly, hypovitaminosis D was more prevalent in the younger groups in the current study. Subgroup analysis by gender in multivariate logistic regression showed that a younger age (26–45 years old) independently predicted hypovitaminosis D in both genders of indoor workers in Taiwan. The finding is consistent with that in recent reports from Korea [36], Thailand [37] and US [22] demonstrating a predisposition to hypovitaminosis D among the younger generation [38]. Although an advanced age is generally associated with an increased risk of hypovitaminosis D attributable to two factors: the decreased efficacy of vitamin D synthesis through UVB-irradiation with aging [39] and less physical activity in the elderly [18], the findings of the present study and those of others [22,36,37,38] appeared to defy such a belief. Some possible explanations include the inclination of the younger generation to stay indoors due to the exponential increase in the utilization of computer technologies both at work and at home [14,40], the use of sunscreen among young people because of cosmetic reasons [38], and a low prevalence of vitamin D intake from vitamin D supplements (<5%) among younger Taiwan people compared to that in the aged population (20%) [13]. The finding of the current study highlighted the widespread hypovitaminosis D problem in the young generation as an urgent health issue that needs to be addressed. Moreover, there is a need to develop and promote strategies to maintain adequate vitamin D status in the young generation.

The incidence of hypovitaminosis D was higher in females than that in males in all age groups. Previous studies have demonstrated a lower vitamin D status in Asian women than that in Asian men [10,11,12], whereas there was no gender difference in serum 25(OH)D in the United States [14,22]. Possible explanations include the general preference of indoor activities among Asian women to avoid sunlight exposure and skin-tanning [11,20,41]. Previous nutrition and health surveys in Taiwan demonstrated a lower mean daily dietary intake of vitamin D in females aged 19–64 in comparison with that in their male counterparts despite a higher consumption of vitamin D supplements in females [13]. Overall, the total vitamin D intake (i.e., food and supplements) in females was lower than that in males [13]. Serum vitamin D status is the result of a complex interaction involving multiple factors including sunlight availability, actual exposure, dietary vitamin D intake and the use of supplements [6,20]. Vitamin D supplementation can be considered to be an alternative to prevent hypovitaminosis D for females. Government should encourage research programs on the impact of hypovitaminosis D on females and individuals with minimal sun exposure.

The present study showed that season (winter/spring) was an independent predictor of hypovitaminosis D for office workers in subtropical Taiwan. The finding is similar to that of a previous study on office workers in subtropical Australia [9]. Interestingly, season was found to be more important than age and gender in determining serum 25(OH)D level for office workers in subtropical Australia [9]. However, age and gender were found to be more important than season in determining serum 25(OH)D level for office workers in subtropical Taiwan. In particular, subgroup analysis by gender in the current study revealed a significant increase in the risk of hypovitaminosis D during winter/spring in males only but not in females. The lack of seasonal difference in serum 25(OH)D levels and the significantly higher incidence of hypovitaminosis D compared with that in males regardless of season among the female population may be partly explained by the fact that Taiwanese females, like those in Hong Kong, Japanese, and Korea, tend to avoid sun exposure by using sunscreen and parasols due to cosmetic concern based on the cultural aesthetic concept [11,20,41]. A longitudinal one-year study among indoor workers also revealed that the monthly change in 25(OH)D in males was more significant than that in females in Japan [42]. Nevertheless, the relative impact of this behavioral factor on 25(OH)D status remains to be elucidated.

Previous studies have demonstrated an inverse correlation between BMI and serum 25(OH)D concentrations in adults, in particular those who are “overweight”(BMI 25 ≤ BMI < 30) or “obese”(BMI≥30) [16,17]. However, compared with those with BMI within the normal range, there was no statistical association between BMI and the risk of hypovitaminosis D in the current study. Consistently, a previous study also showed no significant correlation between the vitamin D status and BMI in underweight individuals and those with normal body weight [16]. In addition, a high prevalence of vitamin D deficiency was found in those with a BMI ≥ 40 (i.e., 32% in women and 46% in men [16]. Therefore, the lack of correlation between BMI and the risk of hypovitaminosis D in the present study may be due to the overall low BMI among indoor workers in subtropical Taiwan (i.e., 14.4 to 36.1 in females, 15.8 to 39.7 in males) with only 7.0% and 25.7% of the subjects being obese and overweight, respectively. Besides, none of them had a BMI ≥ 40.0 (Data not shown). Nevertheless, a previous large-scale study on Taiwanese adults comparing the characteristics of those with vitamin D deficiency and those without showed that the former had a significantly lower mean BMI (23.96 ± 3.99) than that of the latter (24.65 ± 3.72) [26]. However, that study did not focus on indoor workers and no multivariate regression analysis was performed to further elucidate the predictive value of BMI for vitamin D deficiency. When both genders were analyzed separately, neither showed significant association between the risk of hypovitaminosis D and BMI. Possible reasons could be malnutrition resulting from an attempt to maintain an ideal lean body mass in Taiwanese females [41]. Possibly, males who were overweight but not obese were not physically inactive because physical activity was shown to effectively increase vitamin D concentrations [18]. Therefore, overweight males did not have increased risk for hypovitaminosis D (AOR 1.06, 95% CI 0.85, 1.23) in comparison to the normal BMI males.

In our study population, the incidence of hypocalcemia in subjects with hypovitaminosis D was significantly greater than that in subjects with optimal vitamin D status. The mean concentration of corrected total serum calcium levels in subjects with hypovitaminosis D was also significantly lower than that in subjects with optimal vitamin D status. Serum calcium levels are regulated by homeostatic mechanisms involving calcium-sensing receptor, vitamin D and parathyroid hormone. Calcium is absorbed through both transcellular and paracellular pathways across the tight junctions. 1,25-dihydroxyvitamin D can promote paracellular calcium diffusion via up-regulation of tight junction proteins (claudin-2 and -12) in enterocytes through vitamin D receptors [43]. However, intestinal absorption of calcium increases as 25(OH)D rises up but without further increase in calcium absorption above 25(OH)D of 30–32 ng/mL [44]. Intriguingly, the adjusted AUC for hypocalcemia to predict the incidence of hypovitaminosis D was 0.57 indicating no discrimination in the present study. Thus far, the correlation between serum 25(OH)D and corrected total serum calcium was not conclusive. In the elderly, a significant negative correlation between serum 25(OH)D and corrected serum calcium was found [45]. Conversely, there was no significant correlation between serum 25(OH)D and corrected total serum calciumin some reports [46,47]. Besides hypovitaminosis D, causes of hypocalcemia include hypoparathyroidism [48], intestinal malabsorption, magnesium depletion [49] and hyperphosphatemia [30]. High prevalence of hyperphosphatemia in the present study may be a result of the subjects to receive a phosphorus-containing laxative as the preparation for a colonoscopy. During acute hyperphosphatemia, phosphate can bind calcium avidly to cause acute hypocalcemia. Hypocalcemia is common in chronic renal failure because of reduced renal synthesis of 1,25-dihydroxyvitamin D. However, only two patients had eGFR < 30 in this study population. Taken together, total serum calcium levels may not reflect serum vitamin D status. Assumptions about vitamin D status should not be made based on calcium levels. 25(OH)D survey should be requested when hypovitaminosis D is clinically suspected. Thus, there is a need to develop a rapid, reliable, fully automated diagnostic laboratory testing and cost saving assay for 25(OH)D [50].

This study had some limitations. First, the findings were only from a single tertiary care center. However, the hospital provides community care and social service for a population >1.3 million that constituted a large database for the current study. Moreover, we used random sampling technique and paralleled key variables including age, gender and seasons (months) to minimize selection bias. Second, data of vitamin D intake, sun exposure behaviors, drugs such as steroids and physical activity, which are factors known to affect serum 25(OH)D concentration [6,40], were not available in the present retrospective study. Nonetheless, few studies focused on the vitamin D status of indoor office workers in a subtropical Asian region—the strength of the present study. Third, ethnicity has been found to be a predictor of 25(OH)D levels [6,15]. Because only Taiwanese were included in the present study, the findings may not be extrapolated to other ethnic groups. Therefore, further large-scale prospective studies are warranted to determine the relative contributions of environmental factors, behavior differences, and total intake to changes in serum 25(OH)D concentrations of indoor workers living in a subtropical region.

## 5. Conclusions

In conclusion, there was a high prevalence of hypovitaminosis D among indoor office workers in a subtropical region. Younger age, female gender and season (winter/spring) were found to be predictors of hypovitaminosis D in the study population, whereas body-mass index was not a significant factor. The findings underscored the importance of this occupational health issue in subtropical regions. There is, therefore, a need to develop strategies to maintain adequate vitamin D status in indoor workers even in a subtropical region. Future research may include case-control studies comparing the vitamin D status in indoor workers with that in outdoor workers. Despite higher incidence of hypovitaminosis D in indoor workers with hypocalcemia, there was no definite association between vitamin D status and calcium levels in the present study. Assumptions about vitamin D status should not be made based on calcium levels. 25(OH)D survey should be requested when hypovitaminosis D is clinically suspected. Further studies are needed to investigate the association between vitamin D status and calcium levels. Prospective studies are necessary to exact more strong conclusions on these findings.

## Figures and Tables

**Table 1:** Demographic characteristics of indoor workers in subtropical Taiwan.

	Total (*n* = 2880)	Female (*n* = 1440)	Male (*n* = 1440)	*p*
Age, mean (SD), years	46 (11)	46 (11)	46 (11)	1.0
BMI, mean (SD)	24 (4)	22 (4)	25 (4)	0.75
Vitamin D status, *n* (%)				
Optimal (≥30 ng/mL)	1098 (38.1)	405 (28.1)	693 (48.1)	<0.001
Hypovitaminosis D (<30 ng/mL)	1782 (61.9)	1035 (71.9)	747 (51.9)	
25(OH)D, mean (SD), ng/mL	29 (9)	26 (8)	31 (10)	<0.001
25(OH)D in age groups, mean (SD), ng/mL, *n*				
26–35 years	26 (7), 720	23 (7), 360	28 (7), 360	<0.001
36–45 years	27 (8), 720	25 (7), 360	29 (9), 360	<0.001
46–55 years	30 (9), 720	28 (8), 360	31 (9), 360	<0.001
56–65 years	33 (10), 720	30 (8), 360	35 (12), 360	<0.001
25(OH)D in seasons, mean (SD), ng/mL, *n*				
Shorter daylight (Winter/Spring)	28 (9), 1440	26 (7), 720	30 (10), 720	<0.001
Longer daylight (Summer/Autumn)	30 (9), 1440	27 (7), 720	32 (10), 720	<0.001
25(OH)D in BMI groups, mean (SD), ng/mL, *n*				
Obese (≥30.0)	28 (9), 203	27 (8), 57	28 (9), 146	0.63
Overweight (25 ≤ BMI < 30)	29 (9), 741	28 (8), 239	30 (9), 502	0.01
Normal (20 ≤ BMI < 25)	29 (10), 1431	26 (8), 741	31 (10), 690	<0.001
Under weight (<20.0)	27 (8), 505	26 (8), 403	32 (10), 102	<0.001
Corrected total serum calcium concentration, mean (SD), mg/ml	8 (1)	8 (0)	8 (1)	0.33
Serumphosphate concentration, mg/ml	7 (2)	7 (2)	7 (2)	0.19
eGFR	83 (15)	85 (14)	82 (15)	0.65

*n*: number.

**Table 2:** Characteristics of all indoor office workers and indoor workers with hypovitaminosis D or optimal vitamin D levels.

Vitamin D Status	Hypovitaminosis D (<30 ng/mL), *n* = 1782 (61.9%), *n* (%)	Optimal (≥30 ng/mL), *n* = 1098 (38.1%), *n* (%)	Crude OR (95% CI)	Adjusted OR (95% CI)
Gender				
Female (*n* = 1440)	1035 (71.9)	405 (28.1)	2.37 (2.03, 2.77)	2.33 (1.75, 3.09) *
Male (*n* = 1440)	747 (52.7)	693 (47.3)	1.0	1.0
Age group, years				
26–35 (*n* = 720)	543 (75.4)	177 (24.6)	3.65 (2.91, 4.56)	4.32 (2.98, 6.24) *
36–45 (*n* = 720)	506 (70.3)	214 (29.7)	2.81 (2.26, 3.49)	2.82 (1.93, 4.12) *
46–55 (*n* = 720)	404 (56.1)	316 (43.9)	1.52 (1.23, 1.87)	1.50 (1.03, 2.17) *
56–65 (*n* = 720)	329 (45.7)	391 (54.3)	1.0	1.0
Season				
(Winter/Spring) (*n* = 1440)	952 (66.1)	488 (33.9)	1.43 (1.23, 1.67)	1.55 (1.08, 2.22) *
(Summer/Autumn) (*n* = 1440)	830 (57.6)	610 (42.4)	1.0	1.0
BMI (kg/m^2^)				
Obese (≥30.0)	133 (65.5)	70 (34.5)	1.17 (0.86, 1.59)	
Overweight (25 ≤ BMI < 30)	427 (57.6)	314 (42.4)	0.84 (0.70, 1.00)	
Normal (20 ≤ BMI < 25)	886 (61.9)	545 (38.1)	1.0	
Under weight (<20.0)	336 (66.5)	169 (33.5)	1.22 (0.99, 1.51)	

*n*: number. * *p* < 0.05.

**Table 3:** Subgroup analysis of adjusted OR and 95% CI for the risk of hypovitaminosis D in females.

	Females	(*n* = 1440)		
Vitamin D Status	Hypovitaminosis D, *n* = 1035, n (%)	Optimal, *n* = 405, *n* (%)	Crude OR (95% CI)	Adjusted OR (95% CI)
Age group, years				
26–35 (*n* = 360)	308 (86.1)	52 (13.9)	4.74 (3.31, 6.79)	4.05 * (2.35, 6.98)
36–45 (*n* = 360)	292 (81.1)	68 (18.9)	3.44 (2.46, 4.81)	3.22 * (1.81, 5.71)
46–55 (*n* = 360)	235 (65.3)	125 (34.7)	1.50 (1.11, 2.03)	1.67 (1.00, 2.80)
56–65 (*n* = 360)	200 (55.6)	160 (44.4)	1.0	1.0
Season				
(Winter/Spring) (*n* = 720)	538 (74.7)	182 (25.3)	1.33 (1.05, 1.67)	1.12 (0.78, 1.62)
(Summer/Autumn) (*n* = 720)	497 (69.0)	223 (31.0)	1.0	1.0
BMI (kg/m^2^), *n* (%)				
Obese (≥30.0)	39 (68.4)	18 (31.6)	0.84 (0.47, 1.50)	
Overweight (25 ≤ BMI < 30)	169 (70.7)	70 (29.3)	0.94 (0.68, 1.29)	
Normal (20 ≤ BMI < 25)	534 (72.1)	207 (27.9)	1.0	
Under weight (<20.0)	293 (72.7)	110 (27.3)	1.0 (0.79,1.35)	

* *p* < 0.05.

**Table 4:** Subgroup analysis of adjusted OR and 95% CI for the risk of hypovitaminosis D in males.

	Males	(*n* = 1440)		
Vitamin D Status	Hypovitaminosis D, *n* = 747, *n* (%)	Optimal, *n* = 693, *n* (%)	Crude OR (95% CI)	Adjusted OR (95% CI)
Age group, years				
26–35 (*n* = 360)	235 (65.8)	125 (34.2)	3.37 (2.48, 4.57)	4.73 * (2.76, 8.09)
36–45 (*n* = 360)	214 (60.6)	146 (39.4)	2.62 (1.94, 3.55)	2.30 * (1.34, 3.92)
46–55 (*n* = 360)	169 (47.5)	191 (52.5)	1.58 (1.18, 2.14)	1.22 (0.70, 2.13)
56–65 (*n* = 360)	129 (36.9)	231 (63.1)	1.0	1.0
Season				
(Winter/Spring) (*n* = 720)	414 (57.5)	306 (42.5)	1.57 (1.28, 1.94)	1.99 * (1.40, 2.83)
(Summer/Autumn) (*n* = 720)	333 (46.2)	387 (53.8)	1.0	1.0
BMI (kg/m^2^), *n* (%)				
Obese (≥30.0)	94 (64.4)	52 (35.6)	1.74 (1.20, 2.51)	1.27 (0.89, 1.94)
Overweight (2530)	258 (51.4)	244 (48.6)	1.02 (0.81, 1.28)	1.06 (0.85, 1.23)
Normal (20 ≤ BMI < 25)	352 (51.0)	338 (49.0)	1.0	1.0
Under weight (<20.0)	43 (42.2)	59 (57.8)	0.70 (0.46, 1.07)	0.78 (0.57, 1.10)

OR: odds ratio; CI: confidence intervals. Multivariate logistic regression models adjusted for age, season and BMI. * *p* < 0.05.

**Table 5:** Distribution of hypovitaminosis D and optimal vitamin D among indoor workers with hypocalcemia and normal calcium levels.

Corrected Total Serum Calcium Status *	Hypocalcemia (<8.4 mg/mL) (*n* = 1413)	Normal Calcium Levels (8.4~10.4 mg/mL) (*n* = 1467)	*p*	Calcium Levels Mean (SD), mg/mL
Vitamin D status (serum 25(OH)D concentrations), *n* (%)			<0.001	
Deficiency (<20 ng/mL) (*n* = 408)	214 (15.1)	194 (13.2)		8.39 (0.41)
Insufficiency (20–29.9 ng/mL) (*n* = 1374)	716 (50.7)	658 (44.9)		8.39 (0.40)
Optimal (≥30 ng/mL) (*n* = 1098)	483 (34.2)	615 (41.9)		8.47 (0.39)

*n*: number. * None had hypercalcemia. Corrected total serum calcium concentrations was calculated by measured total calcium + (0.8 × (4.0−(albumin))).

## Data Availability

Anonymized data not published within this article will be made available and shared by request from any qualified investigator.

## Abbreviations

AUC	Area under the receiver operating characteristic curve
25(OH)D	25-hydroxyvitamin D
BMI	Body mass index
ELISA	Enzyme linked immunosorbent assay
ICD-9	International Classification of Diseases, Ninth Revision, Clinical Modification
ROC	Curve a receiver operating characteristic curve
